# Comparative Clinical Outcomes of Reconstructive Flap Techniques for Exposed Achilles Tendon: A Prospective Study

**DOI:** 10.7759/cureus.83631

**Published:** 2025-05-07

**Authors:** Vijaykumar Huded, Gireesh Khodnapur, Ajayguru Senthurvelan, Charan Sai Reddy Munugala

**Affiliations:** 1 Hand and Micro Vascular Surgery, Ganga Hospital, Coimbatore, IND; 2 Plastic Surgery, Shri B M Patil Medical College, Hospital, and Research Centre, BLDE (Deemed to Be University), Vijayapura, IND; 3 Orthopedics, Shri B M Patil Medical College, Hospital, and Research Centre, BLDE (Deemed to Be University), Vijayapura, IND

**Keywords:** exposed achilles tendon, lateral calcaneal artery flap, peroneal artery perforator flap, peroneus brevis flap, propeller flap, reverse sural artery flap

## Abstract

Background: Plastic surgeons continue to face considerable challenges while reconstructing exposed Achilles tendon. Several alternatives for repairing such defects include the reverse sural artery flap, peroneus brevis flap, propeller flap, lateral calcaneal artery flap, and peroneal artery perforator flap. However, each of these techniques has its pros and cons. This study compares our clinical experience to various reconstructive techniques for exposed Achilles tendon.

Methods: Fifteen individuals had their exposed Achilles tendon reconstructed between October 2023 and July 2024. These fifteen cases were classified based on the origin of the wound, comorbidities, operational results, and complications, which were analyzed using a prospective study.

Results: The average age of the patients was 40. All 15 patients tolerated the flap procedure well without significant complications; however, one patient with a reverse sural flap developed complete flap necrosis and required secondary split-thickness skin grafting. One more patient had distal tip necrosis and healed with secondary intention. One patient developed an infection, which was resolved by debridement and consecutive dressings. None of the patients had any functional deficits in their lower extremities.

Conclusions: The flap based on the lateral calcaneal artery is the most common and useful flap for coverage of exposed Achilles tendon with respect to aesthetic appearance, good functional outcome, minimal donor morbidity, and time of surgery; it is the flap of choice for small- to medium-sized exposed Achilles. Our study’s second most commonly used flap was based on the sural artery. This flap can cover medium to larger defects. Similarly, other techniques also had their advantages and disadvantages.

## Introduction

The Achilles tendon is one of the longest and strongest tendons in the body. The main function of Achilles tendon is plantar flexion, walking, running, and jumping as it pulls on the heel upon flexion of the calf muscles, acts as a shock absorber while walking, and provides ankle stability [1,2].

Due to its anatomic peculiarities like poor blood supply, being the thickest tendon, absence of synovial sheath, and its 90-degree winding on its path towards the heel, etc., reconstruction and resurfacing of post-traumatic soft tissue defects of the Achilles tendon region is a challenging task for plastic surgeons [3].

Plastic surgeons continue to face challenges in reconstructing exposed Achilles tendon abnormalities, particularly in patients suffering from peripheral vascular disease [4], which can have abnormalities in circulation, and in patients with chronic wounds due to high-intensity trauma [5]. The literature reports a variety of options for covering Achilles tendon soft tissue abnormalities [6].

Many papers have been published regarding the coverage options of the exposed Achilles tendon region. However, the ideal resurfacing options should be stable and thin enough to allow footwear and should provide a gliding surface for the Achilles tendon [7].

Therefore, the main aim of our study is to analyze and determine the suitable reconstruction options for Achilles tendon defects. We also aim to evaluate and assess which flap is better for Achilles tendon defects.

In this work, we report on our experience covering Achilles tendon abnormalities successfully in 15 patients utilizing different flap methods.

## Materials and methods

A prospective study was designed to study 15 patients admitted to the Department of Plastic Surgery at Shri B M Patil Medical College, Hospital, and Research Centre, BLDE (Deemed to Be University), Vijayapura, India, for the period of nine months from October 2023 to July 2024. Data was gathered with proper follow-up of the patients postoperatively at two weeks, three months, and six months. The inclusion criteria include all the patients presenting to our BLDE emergency with exposed Achilles tendon due to loss of skin, injuries to the Achilles tendon region, and delayed cases of Achilles tendon region injuries, which are primarily treated at peripheral hospitals. Exclusion criteria include patients with life-threatening injuries, tendon defects, and fractures.

Age, sex, site and side of the foot affected, treatment involved, and results were all included. The wounds of the patients were cleaned and assessed. Debridement and regular dressing were done until the wound was prepared for the procedure. Regular X-rays were done to rule out any osteomyelitic changes.

Operative technique

Under general or spinal anesthesia, all the patients were placed in supine, prone, or lateral positions according to the location of the wound and the flap used for coverage; a hand-held Doppler was used to detect the perforators and locate the pedicle position. All the surgical procedures were used using loupe magnification (2.5-4.5x) and under tourniquet control.

Flap planning was done according to the planning in reverse method, where a plastic surgeon makes use of a lint or cotton and mocks the transfer of the flap by starting from the defect and working backward to the potential donor site. The choice of flap was determined by the defect, site, size, and nature of the wound.

According to the defect size and location, a lateral calcaneal artery flap was done in four patients (Figures 1-5), a peninsular reverse sural artery flap was done in four patients (Figures 6-7), an islanded reverse sural artery flap, a peroneus brevis (Figures 8-10), a peroneal artery perforator flap was done in four patients (Figures 11-12), and a posterior tibial artery-based propeller flap were done in one patient each.

**Figure 1:**
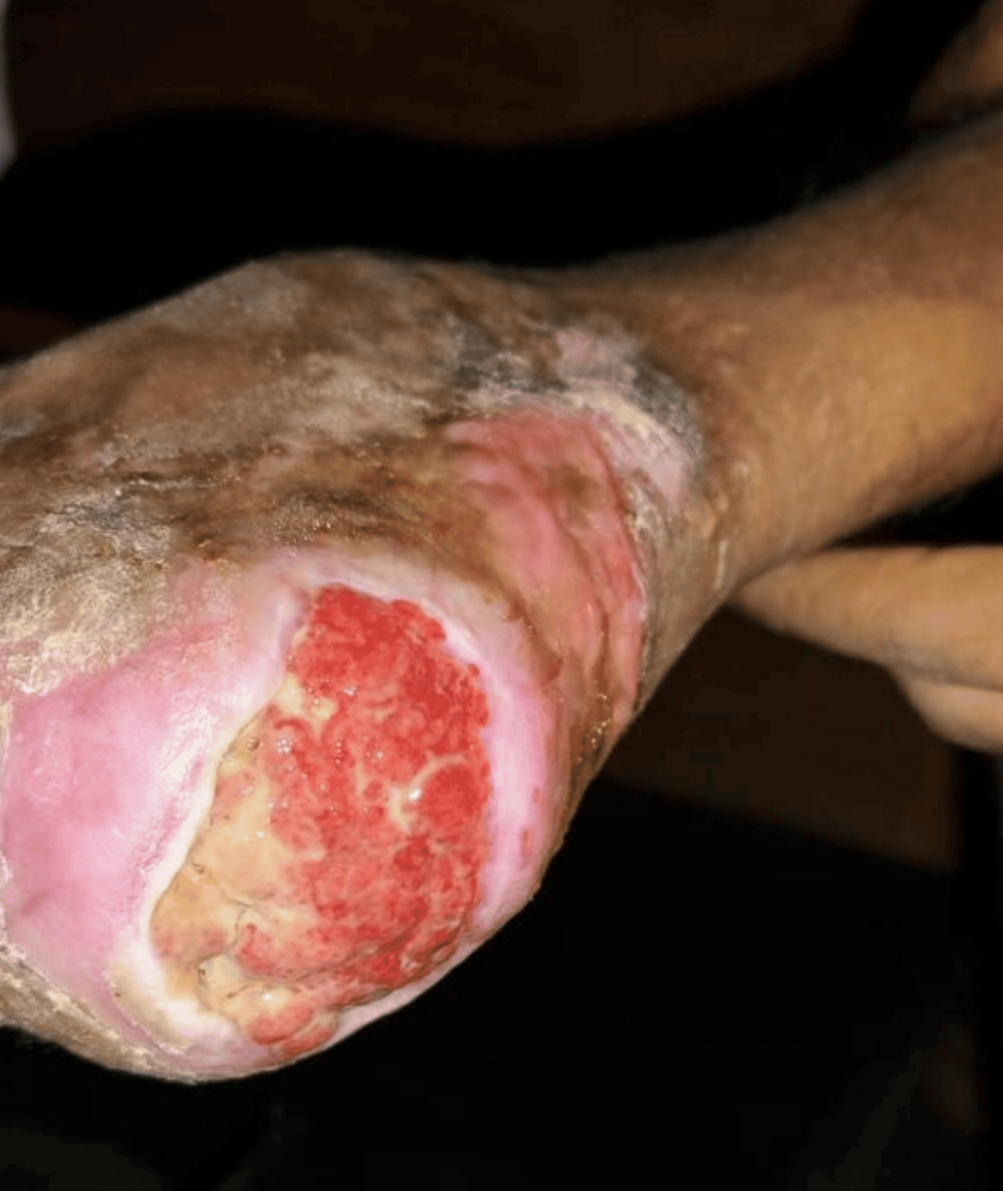
Preoperative lateral calcaneal artery flap

**Figure 2:**
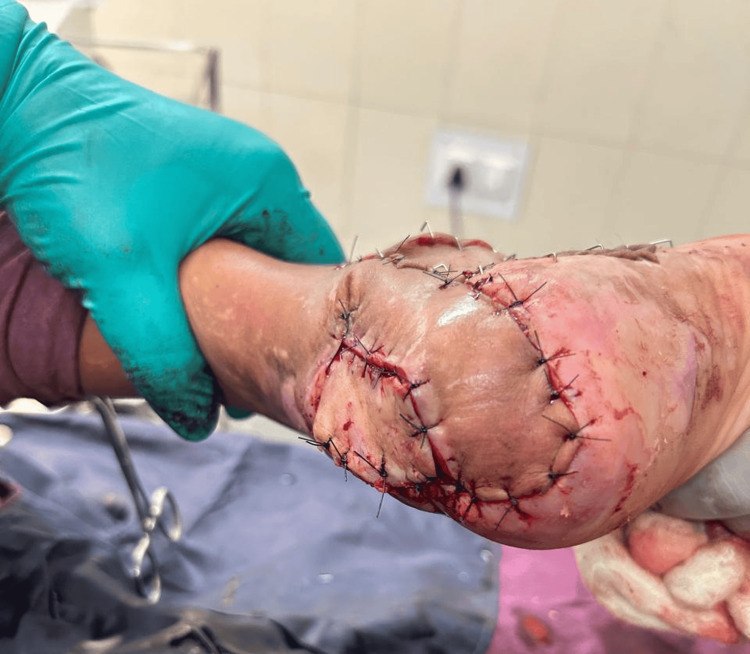
Postoperative lateral calcaneal artery flap

**Figure 3:**
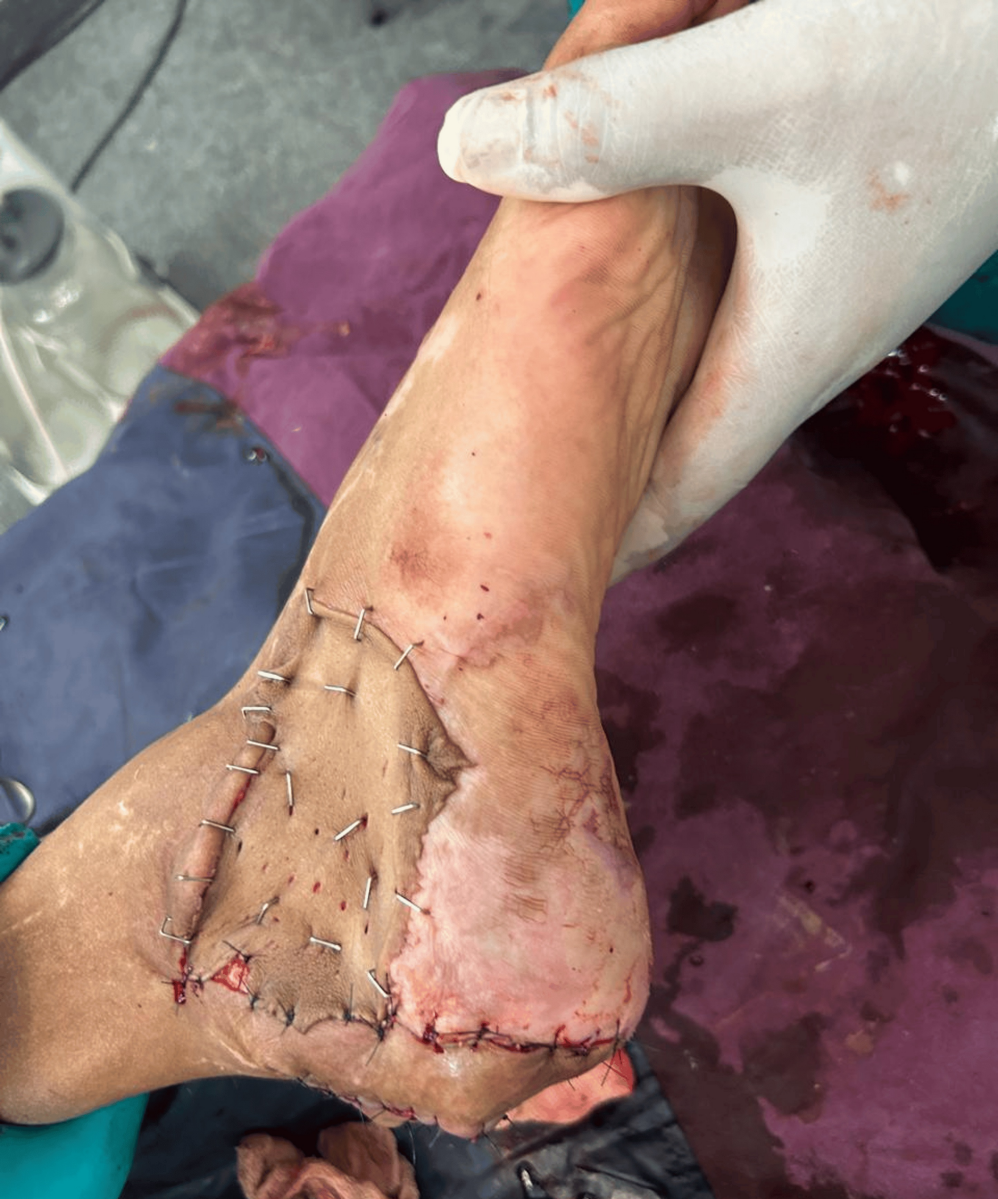
Postoperative donor defect of lateral calcaneal artery flap with split-thickness skin graft

**Figure 4:**
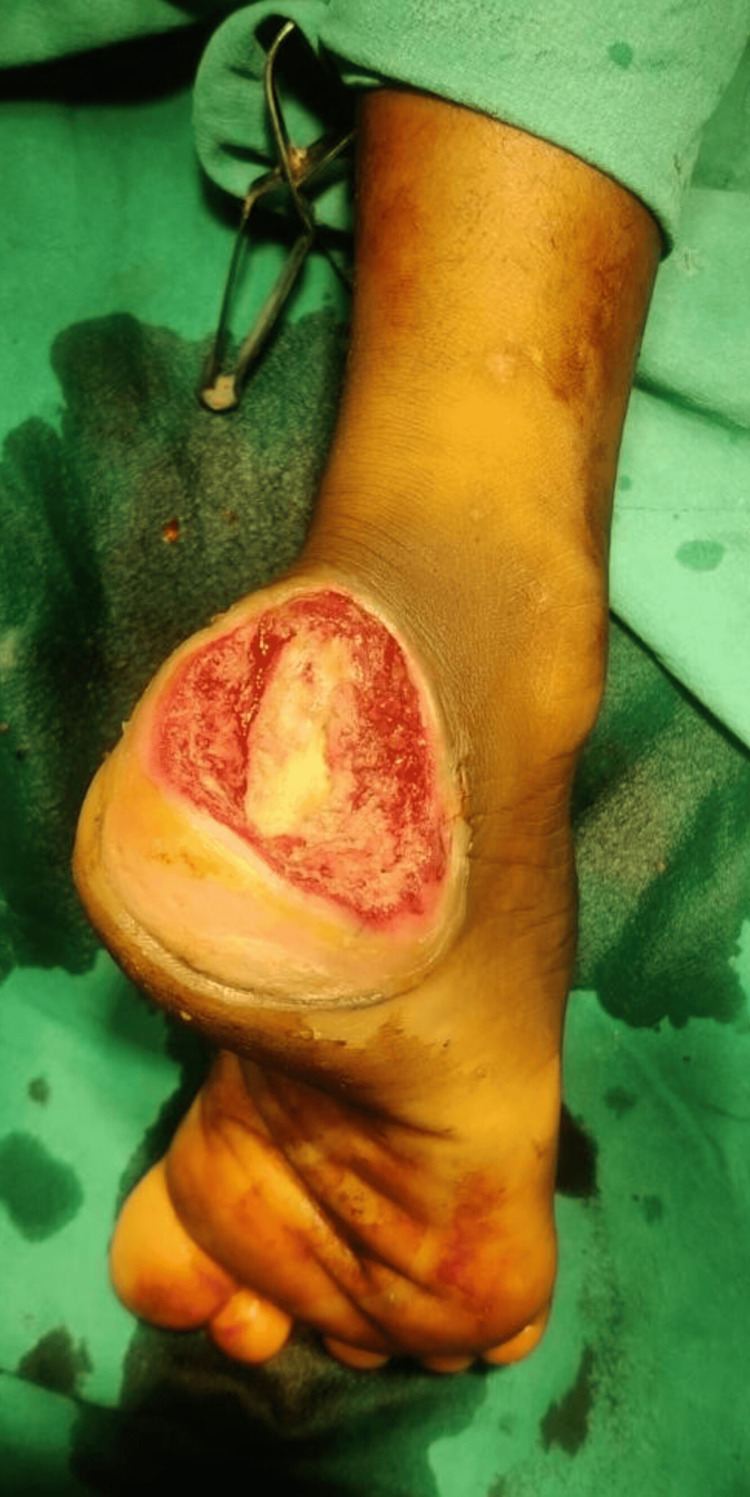
Preoperative lateral calcaneal artery flap

**Figure 5:**
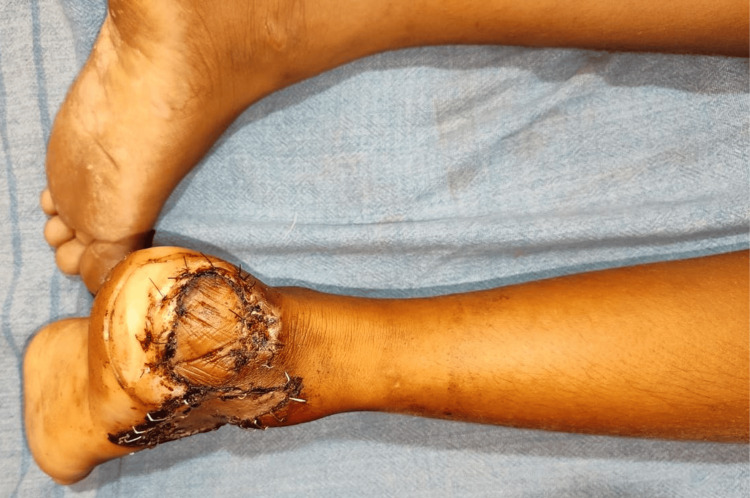
Postoperative lateral calcaneal artery flap

**Figure 6:**
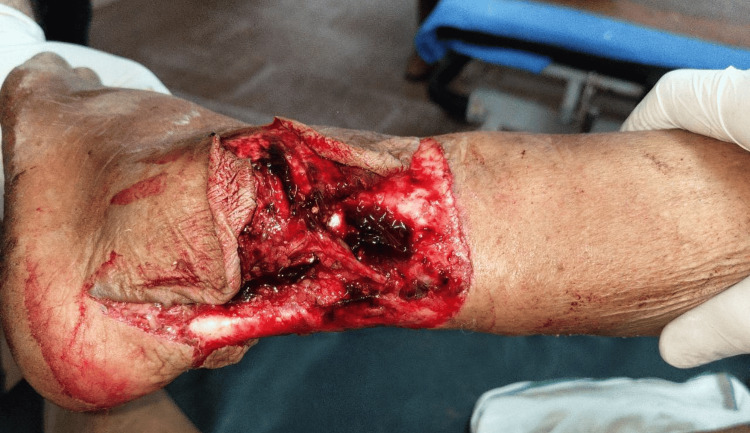
Preoperative reverse sural artery flap

**Figure 7:**
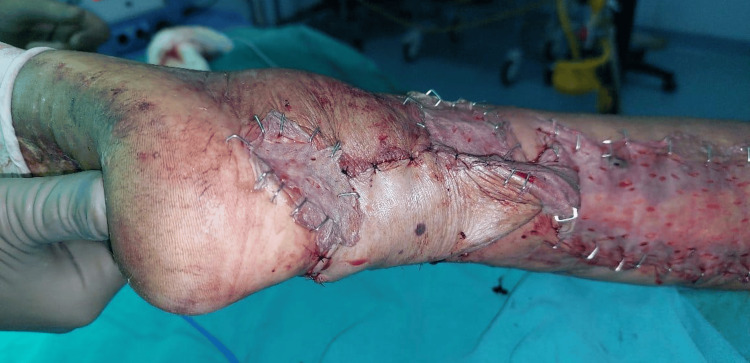
Postoperative reverse sural artery flap

**Figure 8:**
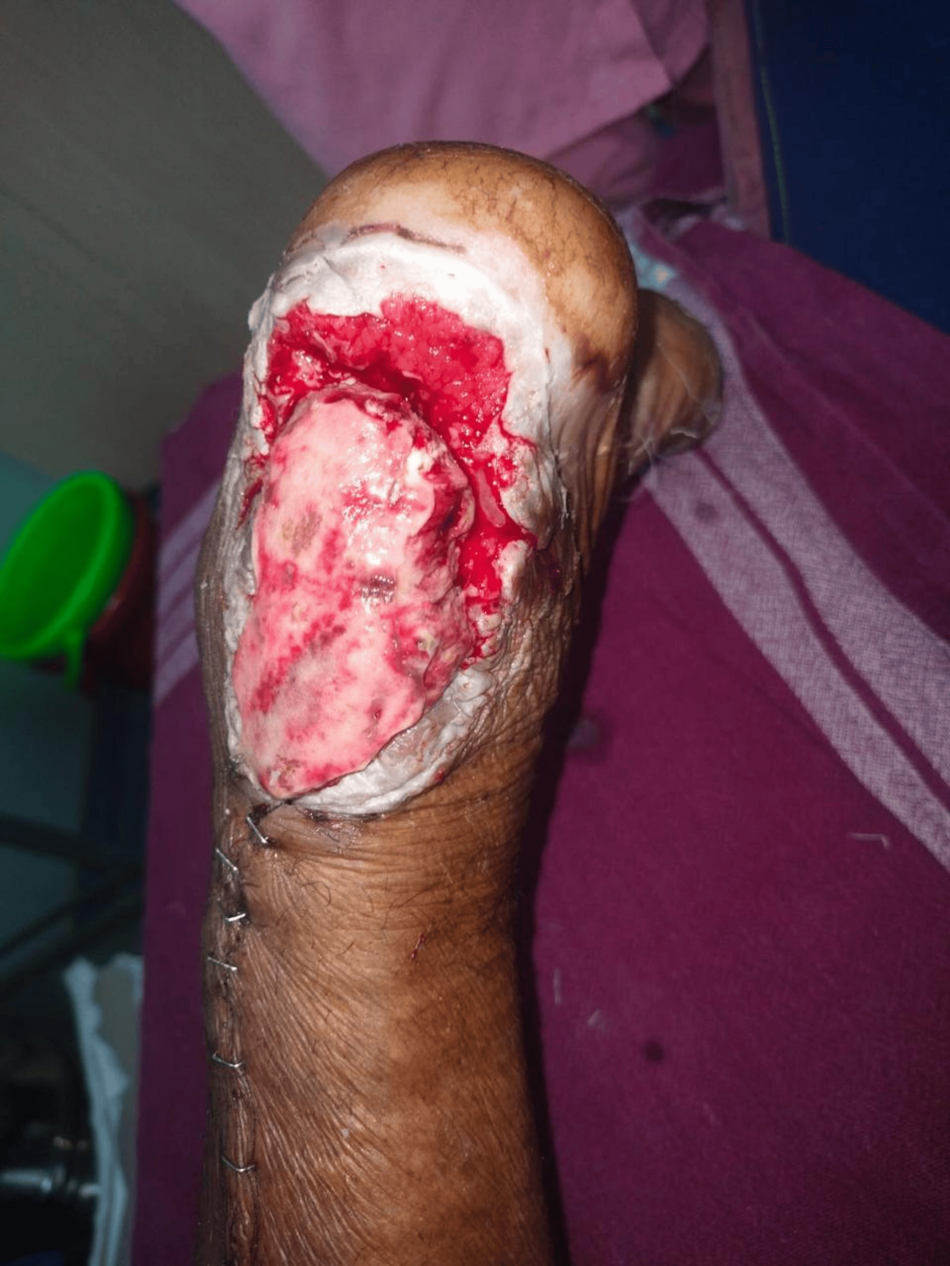
Preoperative peroneus brevis muscle flap

**Figure 9:**
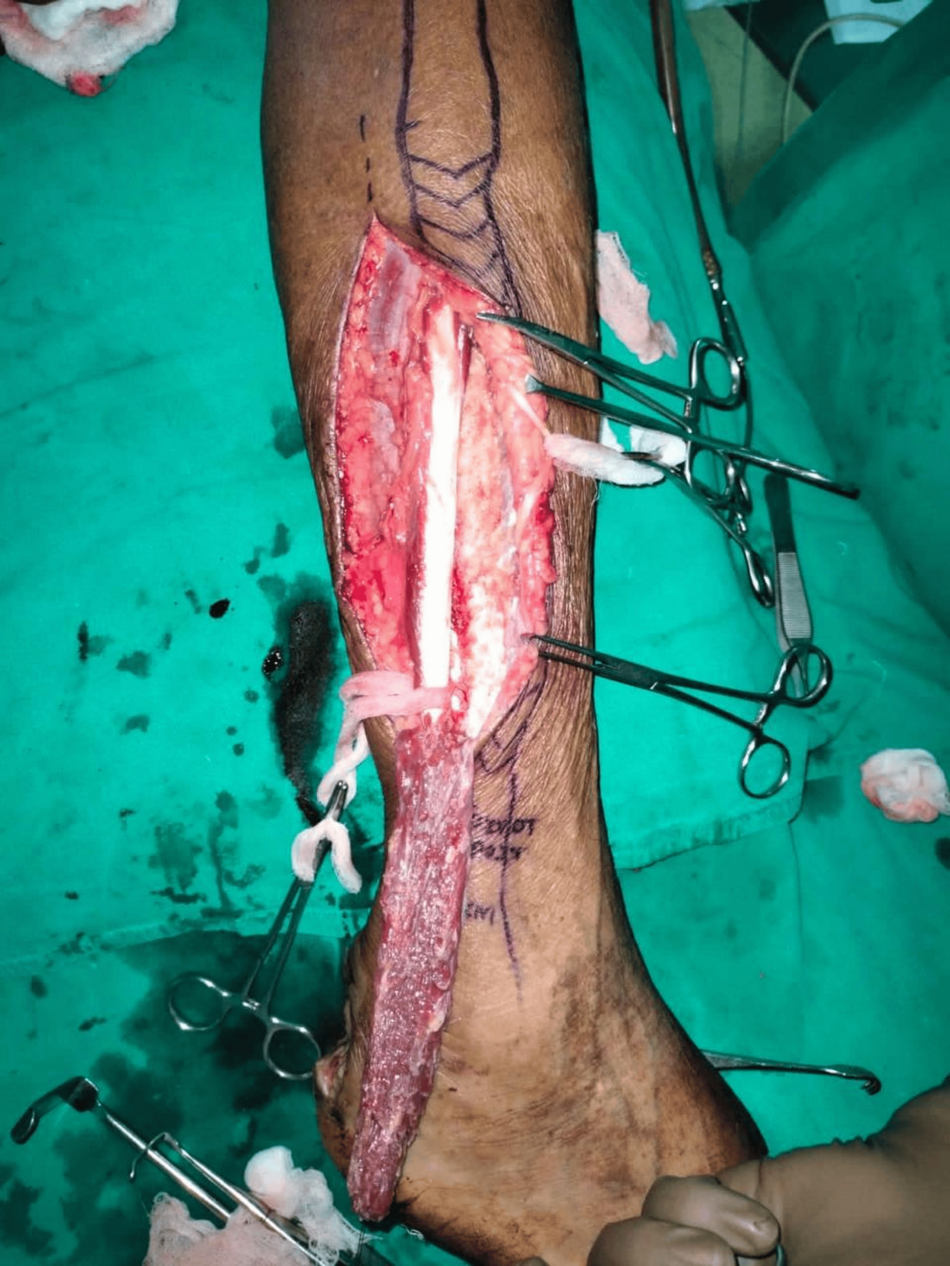
Intraoperative peroneus brevis artery flap

**Figure 10:**
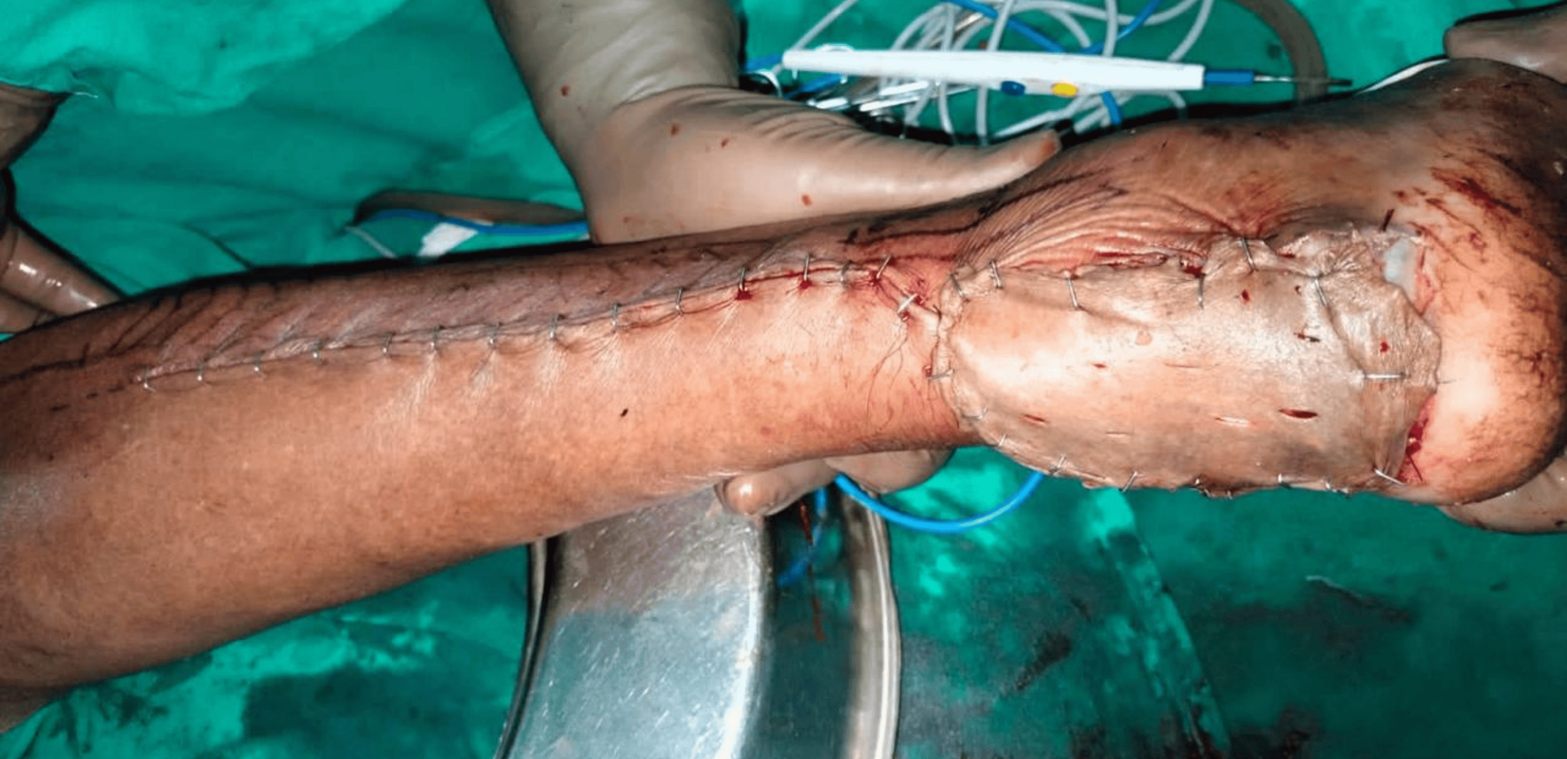
Postoperative peroneus brevis artery flap

**Figure 11:**
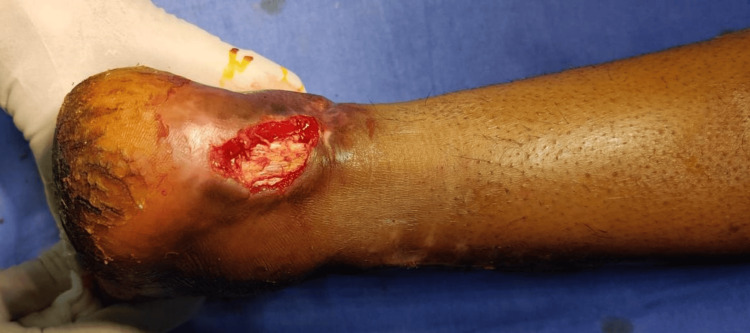
Preoperative peroneal artery perforator based flap

**Figure 12:**
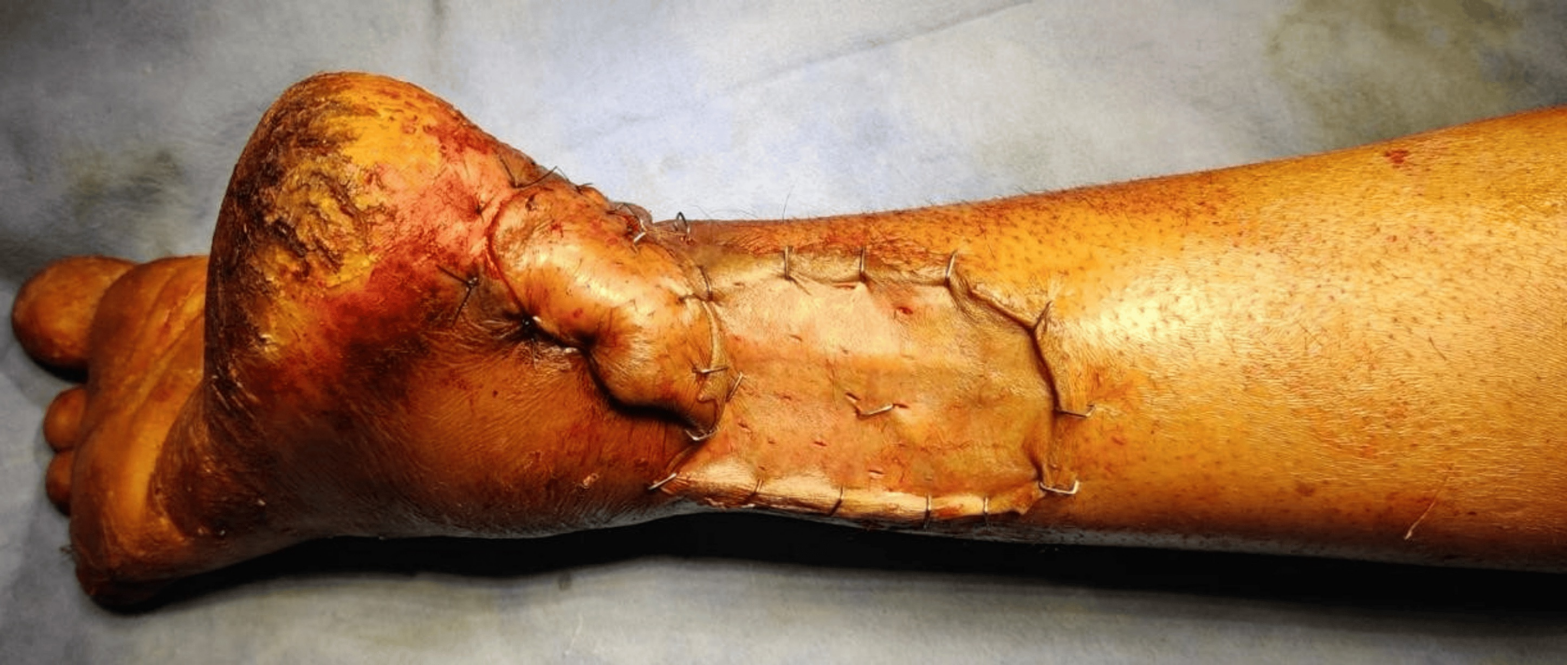
Postoperative peroneal artery perforator based flap

The color and pinpricks of the flap were used to frequently assess its vascularity. Peripheral flap necrosis was treated by debriding the affected area and then closing the wound with a secondary purpose. During their postoperative follow-up appointments, patients had their foot functionality assessed for movements.

Postoperative care

A soft bandage was used to cover the flap site, and a small area was left exposed to check the flap viability, flap condition, infection, walking activities, and thinning of flaps in case of bulky flaps. The operated limb was kept elevated to facilitate venous drainage and reduce edema and pain. The flap was monitored regularly for any vascular insufficiency. The first dressing was done on the first operative day, broad-spectrum antibiotics were administered for seven days postoperatively, and follow-up was done for at least a six-month period.

## Results

The average age of the patients was 40 years. The mean interval period from the time of injury to presentation was seven days (range: 5-9 days). Initially, patients were treated by orthopedic surgeons and later referred to plastic surgeons. Immediate coverage was not done due to extensive infection, the clinical condition of the patient at the time of presentation, and also due to the delayed consent from the patient and the patient attenders, who generally seek intervention from hospitals in metro cities, which explains the reason for the delay. Fourteen patients survived the flap with no serious problems; however, one patient with reverse sural flap had complete necrosis, and a secondary split-thickness skin graft was done. One more patient had distal tip necrosis and healed it with secondary intention. One patient developed an infection, which was resolved by debridement and consecutive dressings with antimicrobial therapy, which was given according to the culture and sensitivity study. None of the patients had any other functional deficits in their lower limbs.

In our study, lateral calcaneal artery flap was done in four (26.67%) patients (Figures 1-5), peninsular reverse sural artery flap was done in four (26.67%) patients (Figures 6-7), islanded reverse sural artery flap was done in one (6.67%) patient, peroneus brevis muscle graft was done in one (6.67%) patient (Figures 8-10), peroneal artery perforator flap was done in four (26.67%) patients (Figures 11-12), and posterior tibial artery flap was done in one (6.67%) patient.

Of the fifteen cases, nine were right-sided, and the rest six cases were left-sided injuries. The details are listed in Table 1.

**Table 1:** Description of study details

Serial number	Age/sex	Mode of injury	Occupation	Comorbidities	Diagnosis	Procedure	Complications	Re-intervention
1	24/M	Road traffic accident	Daily wager	-	Exposed Achilles tendon with heel defect (distal most part and posterior and lateral defects of hind foot)	Lateral calcaneal artery flap	-	-
2	8/M	Road traffic accident	Schooling	-	Exposed Achilles tendon (distal most part and posterior and lateral defects of hind foot)	Lateral calcaneal artery flap	-	-
3	42/M	Road traffic accident	Farmer	-	Exposed Achilles tendon (distal most part and posterior and lateral defects of hind foot)	Lateral calcaneal artery flap	Distal tip necrosis	Healed by secondary intention
4	35/M	Road traffic accident	Driver	-	Exposed Achilles tendon with heel defect (on lateral aspect of heel)	Peroneal artery flap	-	-
5	50/M	Road traffic accident	Farmer	Diabetic	Exposed Achilles tendon (distal most part and posterior and lateral defects of hind foot)	Lateral calcaneal artery flap	-	-
6	35/F	Road traffic accident	Housewife	-	Exposed Achilles tendon (proximal and mid Achilles tendon defects)	Islanded reverse sural artery flap	-	-
7	60/M	Road traffic accident	Farmer	Hypertensive	Exposed Achilles tendon	Peroneus brevis flap	-	-
8	40/M	Road traffic accident	Tailor	-	Exposed Achilles tendon	Reverse sural flap	Infection	-
9	50/M	Road traffic accident	Butcher	Diabetic	Exposed Achilles tendon	Propeller flap (PTA)	-	-
10	35/F	Pothole injury	Housewife	-	Exposed Achilles tendon	Reverse sural flap	Complete necrosis	Split-thickness skin graft
11	41/M	Road traffic accident	Weaver	-	Exposed Achilles tendon	Reverse sural flap	-	-
12	48/M	Road traffic accident	Farmer	-	Exposed Achilles tendon	Reverse sural flap	-	-
13	50/M	Road traffic accident	Daily wager	-	Exposed Achilles tendon	Peroneal artery flap	-	-
14	37/M	Road traffic accident	Daily wager	-	Exposed Achilles tendon (on lateral aspect of heel)	Peroneal artery flap	-	-
15	53/M	Road traffic accident	Driver	Diabetic	Exposed Achilles tendon (on lateral aspect of heel)	Peroneal artery flap	-	-

## Discussion

Reconstruction is frequently complicated by Achilles tendon site abnormalities due to their highly functional nature, poor vascular supply, tendinous/bony substrate, and constant movements, and for the very reasons, conservative care is insufficient. The use of grafts (full-thickness or split-thickness) often results in unsightly and functionally unsatisfactory outcomes, including intermittent hypertrophy and scar contracture. Free flap transfers, however, are technically difficult, necessitating a great deal of microsurgical skill and a lengthy surgical procedure, with a high risk of severe perioperative morbidity.

The aim of reconstructive surgery is to replace "like with like" wherever possible and to have flexible soft tissue covering to aid with skeletal repair [8]. To be able to walk, one must maintain both motor and sensory abilities. Tissue loss, location of the defect, size, vascular pedicle length required, and condition of the wound should be taken into account while choosing the optimum reconstructive procedure. The foot is a difficult region to repair and is vulnerable to illness and trauma. To achieve the best practical and cosmetic results, it is best to think about foot rebuilding by subunits. Due to their traumatic origin in the younger population, tissues with sufficient blood supply, high flexibility, and sensibility are needed for the reconstruction of exposed Achilles tendon lesions [9]; otherwise, morbidity may result.

The lateral calcaneal artery flap is a flap for covering posterior heel defects; it is mainly based on the lateral calcaneal artery. This is one of the feasible options for flap cover over the hindfoot [10]. This flap has a number of benefits, including a high rate of success, less postoperative discomfort, adequate coverage of soft tissue, and a positive functional outcome (Figures 1-5). Alam et al.'s [11] study found that the lateral calcaneal artery flap [12] is superior to other reconstruction techniques for posterior heel and tendon Achilles deficits [13] in terms of function, donor area morbidity, and surgical time.

Unlike other authors who performed various microvascular tissue transfers and local flaps, we solely used reverse sural artery flaps (Figures 4-5) [14] for the coverage of deeper-grade injuries [15]. The wounds over the medial malleolus, heel, midfoot, and proximal defects benefit from reversal sural artery flaps. According to Farooq et al., the outcomes are similar in these situations, highlighting the significance of reverse sural artery flap [16]. This procedure is also a good choice in resource-constrained environments and operating rooms without microvascular tissue transfer capabilities.

Flap survival was complete and accomplished without major complications in 12-patient research by Ahn et al. using a pedicle graft based on a peroneal artery perforator; two patients had mild wound dehiscence. Still, these wounds responded favorably to conservative care and later debridement (Figures 11-12) [17]. There were no functional deficiencies in any of the patients' lower limbs.

One patient with a moderate defect was covered with a distally based peroneus brevis muscle flap (Figures 8-10) [18]. The main advantage of the peroneus brevis muscle flap was its technical simplicity and coverage of moderate to large defects involving the heel and anterior aspect of the ankle joint, the only disadvantage being ankle stiffness. According to a study done by Lorenzetti et al., a distally based peroneus brevis muscle flap was used for reconstructing the defects involving the exposed Achilles tendon, heel, and anterior aspect of the ankle [18].

In patients with diabetes who had delayed healing patterns, with strict glycemic control and wound care, the wound was healed properly.

The limitations of our study include a relatively small sample size and poor patient turnover for follow-ups. Additionally, we did not perform free flaps due to manpower limitations.

## Conclusions

The flap based on the lateral calcaneal artery is the most common and useful flap for coverage of exposed Achilles tendon with respect to aesthetic appearance, good functional outcome, minimal donor morbidity, and time of surgery; it is the flap of choice for small- to medium-sized exposed Achilles. It is resistant to atherosclerosis, which is very helpful in treating patients with diabetes. The downside of this procedure is that it is technically challenging and is performed by skilled surgeons for moderate to large defects. Our study’s second most commonly used flap was based on the reverse sural artery. This flap can cover medium to larger defects. The main disadvantage of this procedure is that it causes venous congestion due to the arc of rotation. Similarly, other techniques also had their advantages and disadvantages.

To conclude, the lateral calcaneal artery flap is the most commonly used flap for distal-most Achilles tendon defects and hindfoot defects; the reverse sural artery flap is second most commonly used for moderate to large Achilles tendon region defects, the peroneus brevis flap is for moderate lateral Achilles tendon defects, and the propeller (peroneal) based artery is for smaller lateral Achilles tendon defects.
